# ﻿Revision of the “ *Chloritisdelibrata* (Benson, 1836)” group (Gastropoda, Stylommatophora, Camaenidae)

**DOI:** 10.3897/zookeys.1086.77180

**Published:** 2022-02-15

**Authors:** Barna Páll-Gergely, Jonathan D. Ablett, Márton Szabó, Eike Neubert

**Affiliations:** 1 Plant Protection Institute, Centre for Agricultural Research, Herman Ottó Street 15, Budapest, H-1022, Hungary Plant Protection Institute, Centre for Agricultural Research Budapest Hungary; 2 Mollusca Section, Invertebrates Division, Department of Life Sciences, The Natural History Museums, London SW7 5BD, UK The Natural History Museums London United Kingdom; 3 Hungarian Natural History Museum, Department of Paleontology and Geology, Ludovika tér 2, Budapest 1083, Hungary Hungarian Natural History Museum Budapest Hungary; 4 Natural History Museum of the Burgergemeinde Bern, Bernastr. 15, CH-3005 Berne, Switzerland Natural History Museum of the Burgergemeinde Bern Bern Switzerland; 5 Institute of Ecology and Evolution, University of Bern, 3012 Bern, Switzerland University of Bern Bern Switzerland

**Keywords:** *Bouchetcamaena*, *Burmochloritis*, conchology, India, Myanmar, new combinations, new species, shell morphology, systematics, taxonomy

## Abstract

*Chloritisdelibrata* (Benson, 1836), known from northeastern India, was believed to have three varietal forms, sometimes mentioned as subspecies: C.delibratavar.khasiensis (Nevill, 1877) and C.delibratavar.fasciata (Godwin-Austen, 1875) from the Khasi Hills, India, and C.delibratavar.procumbens (Gould, 1844) from Dawei in Myanmar. The reproductive anatomy of the latter form is known and does not match with those of any continental camaenid genera, but does with that of the newly examined *Chloritisplatytropis* Möllendorff, 1894 from Thailand. The latter species is conchologically similar to *Bouchetcamaenahuberi* Thach, 2018 (synonym of *Helixfouresi* Morlet, 1886), which is the type species of the genus *Bouchetcamaena* Thach, 2018. Thus, *Bouchetcamaena* can provisionally host the entire Chloritisdelibrata -group with the exception of var. fasciata, which is transferred to *Burmochloritis* Godwin-Austen, 1920 due to the multiple reddish bands on its shell. The examination of shells deposited in the Natural History Museum, London revealed that seven morphologically distinguishable forms are present, which are accepted here as representing distinct species. Four new species are described from India: *Bouchetcamaenafoveata* Páll-Gergely **sp. nov.**, *B.fusca* Páll-Gergely **sp. nov.**, *B.raripila* Páll-Gergely **sp. nov.**, and *B.subdelibrata* Páll-Gergely **sp. nov.**

## ﻿Introduction

Chloritis (Trichochloritis) delibrata (Benson, 1836) was considered to be a variable camaenid species inhabiting a relatively large area from Assam in India to Dawei (= Tavoy) in Myanmar (Stoliczka 1871; Gude 1914). The distance between these two sites is approximately 1500 km as the crow flies. The last overview of this species was published over a century ago in the Fauna of British India by Gude (1914) who listed three varieties: var. khasiensis (Nevill, 1877) and var. fasciata (Godwin-Austen, 1875) from the Khasi Hills, India, and var. procumbens (Gould, 1844) from Tavoy, Myanmar. The two Indian varieties were listed as subspecies of *Chloritisdelibrata* in the latest Indian checklist (Ramakrishna et al. 2010).

Examination of specimens assigned to *Chloritisdelibrata* and its forms in the Natural History Museum, London, revealed that at least seven species can be distinguished based on the shape the shell and, most importantly, its fine sculpture. Thus, we here give an overview of the *C.delibrata* group, and describe the morphologically recognisable, distinct entities as species.

### ﻿Generic position

**Figure 1.**
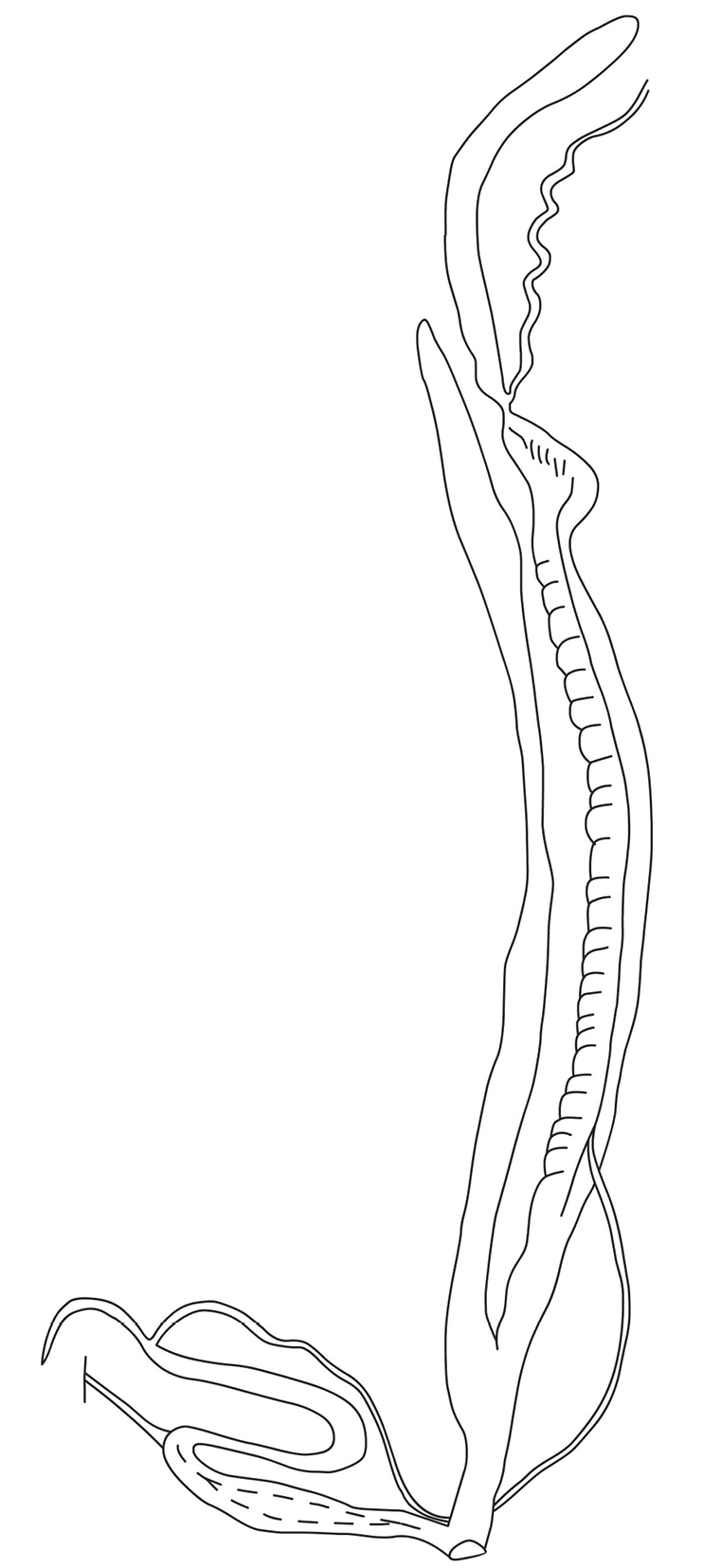
Reproductive anatomy of Chloritisdelibratavar.procumbens (Gould, 1844). Redrawn from Stoliczka (1871).

Placing the *Chloritisdelibrata*-group in its appropriate genus turned out to be challenging. The morphology of the jaw and the radular teeth, along with the outer characters of the reproductive anatomy of “var. procumbens” from Moulmein were described by Stoliczka (1871) [redrawn by Pilsbry (1894) and in this manuscript (Fig. 1)]. Based on these descriptions, the penis is spindle-shaped, the epiphallus is longer than the penis, slender, cylindrical, the retractor muscle inserts at the penis-epiphallus transition, and there is a slender, pointed, moderately short flagellum. We needed to examine the possible placement of the *Chloritisdelibrata*-group in *Chloritis* Beck, 1837, *Trichochloritis* Pilsbry, 1891, and *Trachia* Martens, 1860, since this species complex had previously been placed in those genera, along with *Bellatrachia* Schileyko, 2018, *Bouchetcamaena* Thach, 2018, *Burmochloritis* Godwin-Austen, 1920, *Neotrachia* Schileyko, 2018, *Planispira* Beck, 1837, *Satsuma* A. Adams, 1868, and *Sinochloritis* M. Wu & Z. Chen, 2019, which inhabit the same or adjacent geographic areas. The key traits of the reproductive anatomy are summarized in Table 1.

**Table 1.** Most important character states of camaenid genera relevant for this study.

	References	Penis	Inner structure of the penis	Penial sheath	Epiphallus	Insertion of retractor muscle	Penial caecum	Flagellum	Additional organ
*Bouchetcamaena* Thach, 2018	this study	long, apically thickened	parallel folds and a vestigial verge	absent	long, cylindrical	on distal epiphallus	absent	long, slender	absent
Continental “*Chloritis*”	Sutcharit and Panha (2010), Páll-Gergely et al. (2020)	long, apically thickened	parallel folds and a large verge	absent	long, cylindrical	on distal epiphallus	absent	medium length, gradually becoming slender	absent
*Neotrachia* Schileyko, 2018	Schileyko (2018)	short, thick	longitudinal pilasters, broken into series of tubercles	absent	swollen, ovoid	penis-epiphallus transition	absent	medium length, gradually becoming slender	absent
*Trachia* Martens, 1860 (based on *T.vittata*)	Schileyko (2003)	short, swollen	chaotically arranged pilasters	covers entire penis	rather short, thick	incorporated into penial sheath	absent	rather long, gradually becoming slender	–
*Trichochloritis* Pilsbry, 1891	Collinge (1903), Páll-Gergely and Neubert (2019)	long, apically thickened	unknown	absent	long, cylindrical	on distal epiphallus	moderately long, slender	very short, pointed	absent
*Burmochloritis* Godwin-Austen, 1920	Godwin-Austen (1920), unpublished information	long, thick, cylindrical	wavy folds, verge absent	absent	long, cylindrical	bounds penis and epiphallus at some distance from their junction	short, pointed	long, slender	long, cylindrical, derives from wall of vagina
*Satsuma* A. Adams, 1868	Wang et al. (2014), Zhang et al. (2020)	long, cylindrical	wavy folds, verge absent	absent	long, cylindrical	on distal epiphallus	well-developed, tapering	long to short	absent
*Sinochloritis* M. Wu & Z. Chen, 2019	Wu & Chen, 2019	thick, cylindrical	parallel folds, verge absent	absent	long, cylindrical	on distal epiphallus, and also covers proximal part of penis	large, internally with “peach shaped epiphallic papilla”	long, slender, tapering	absent
*Bellatrachia* Schileyko, 2018	Schileyko (2018), Páll-Gergely and Neubert (2019)	long, cylindrical	parallel folds	absent	long, cylindrical	penis-epiphallus transition	absent	thick, somewhat swollen, with slender tip	absent
*Planispira* Beck, 1837	Schileyko (2003)	short, apically thickened	folds and an ovoid, large verge	absent	long, cylindrical	middle of epiphallus	absent	short, conical	absent

The anatomy of the type species of *Trichochloritis* (*Helixbreviseta* Pfeiffer, 1862) is known based on Stoliczka (1873) (see also Páll-Gergely and Neubert 2019). The most obvious difference is the presence of a slender but relatively long penial caecum, which is absent in C.delibratavar.procumbens. Further differences include the following: flagellum shorter, vagina longer in *Trichochloritis*, and bursa of bursa copulatrix more ovoid, less elongated.

Some species of *Satsuma* (type species: *Helixjaponica* L. Pfeiffer, 1847; SD, Kuroda and Habe 1949) from China are similar to the *delibrata*-group in terms of the thin shell with a single band, but *Satsuma* is characterized by a well-developed penial caecum (Wang et al. 2014; Zhang et al. 2019). We note that those Chinese *Satsuma* species possess a short, vestigial flagellum, which is well developed, rather long in the type species of *Satsuma* (see Emura 1933; Azuma 1995; unpublished information); thus, the generic status of the Chinese *Satsuma* species requires a revision.

*Sinochloritis*, known only from the Chinese Sichuan Province, also possesses a large penial caecum. Thus, we do not consider the *C.delibrata*-group to belong to any of those three genera (*Trichochloritis*, *Satsuma*, *Sinochloritis*).

The type species of *Trachia* Martens, 1860 (*Helixasperella* L. Pfeiffer, 1846) is not known anatomically. The anatomy of *Trachiavittata* (O. F. Müller, 1774) was described by Schileyko (2003), who revealed that it is entirely different from that of *Chloritisdelibrata*, since the retractor muscle joins the fibrous sheath around the penis. Later, Schileyko (2018) claimed that since the shell of *Trachiaasperella* is similar to that of “*Helix*” *delibrata*, their anatomy is also probably similar, and thus, classified *C.delibrata* in *Trachia*. However, *Trachiavittata* possesses multiple spiral bands, whereas only a single band is present in the *delibrata*-group. More importantly, *Trachiavittata* is known from central and southern India (Gude 1904; Mitra et al. 2004), whereas the *delibrata*-group is restricted to the areas southeast of the Himalaya, a biogeographically very distinct region. Moreover, most other species named by Schileyko (2018) as possible *Trachia* species also inhabit the Indian subcontinent. Thus, it is improbable that *Trachiavittata* and the species of the *C.delibrata*-group would belong to the same genus.

*Bellatrachia* differs from Stoliczka’s (1871) drawing of C.delibratavar.procumbens by the much longer penis and epiphallus, the well-developed flagellum, and the thickened base of the bursa copulatrix.

*Burmochloritis* Godwin-Austen, 1920 (type species: *Burmochloritiskengtungensis* Godwin-Austen, 1920, OD) possesses a long flagellum, a well-developed, large penial caecum is, and an additional organ (homologous with dart sac?) originating from the wall of the vagina (Godwin-Austen 1920; and unpublished information).

The genital anatomy of *Neotrachia* is very different from that of C.delibratavar.procumbens due to its short penis and swollen epiphallus (Schileyko 2018).

The type species of *Planispira* Beck, 1837 (*Helixzonaria* Linnaeus, 1767) was redescribed by Schileyko (2003) as having penis swollen and epiphallus relatively short and thick (spindle-shaped penis and long, slender epiphallus in *C.delibrata*), and stalk or bursa copulatrix very long, convoluted.

This makes it improbable that the *C.delibrata*-group belongs to any of the genera *Bellatrachia*, *Burmochloritis*, *Neotrachia*, and *Planispira*.

The type species of the genus *Chloritis* is *Helixungulina* Linnaeus, 1758 (SD, Gray 1847), which was described without stating the type locality. Subsequently it turned out to inhabit Indonesia (Zilch 1966). Although the anatomy of *Chloritisungulina* is unknown, it is highly unlikely that the *Chloritisdelibrata*-group would belong to the same genus due to the geographical separation of the two species. Some continental (Thailand, Vietnam) species are classified in *Chloritis*, and their genitalia largely agree with Stoliczka’s drawing (Sutcharit and Panha 2010; Páll-Gergely and Neubert 2019; Páll-Gergely et al. 2020). However, the presence of a penial caecum distinguishes continental *Chloritis* from *Bouchetcamaena* (see below).

**Figure 2.**
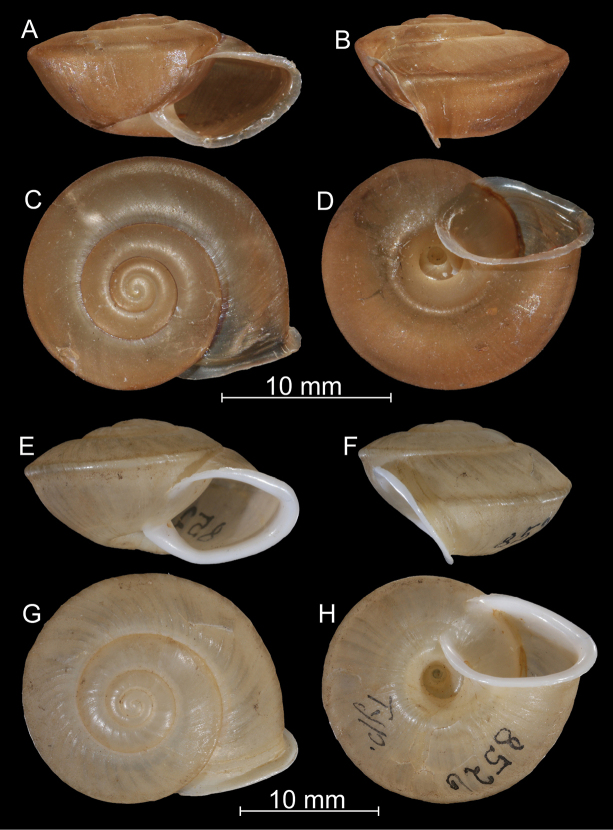
*Bouchetcamaenaplatytropis* Möllendorff, 1894 **A–D** anatomically examined specimen (ZMH 51934) **E–H** lectotype (SMF 8526). All photos: B. Páll-Gergely.

**Figure 3.**
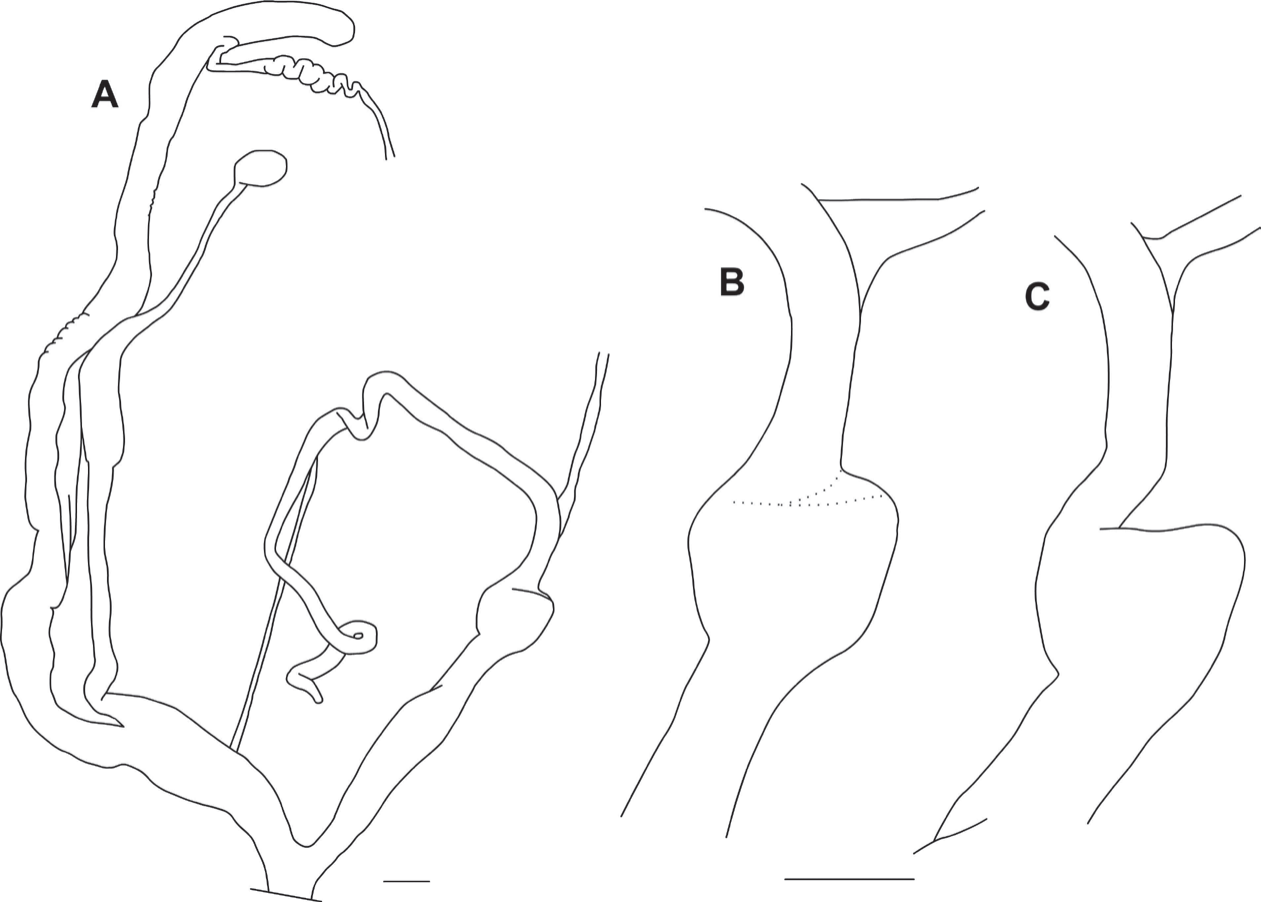
Reproductive anatomy of *Bouchetcamaenaplatytropis* Möllendorff, 1894 (ZMH 51934) **A** entire genitalia **B** penis before cutting the weak fibres connecting proximal end of penis to distal part of epiphallus; **C** after removing the weak fibres. Scales represent 1 mm.

**Figure 4.**
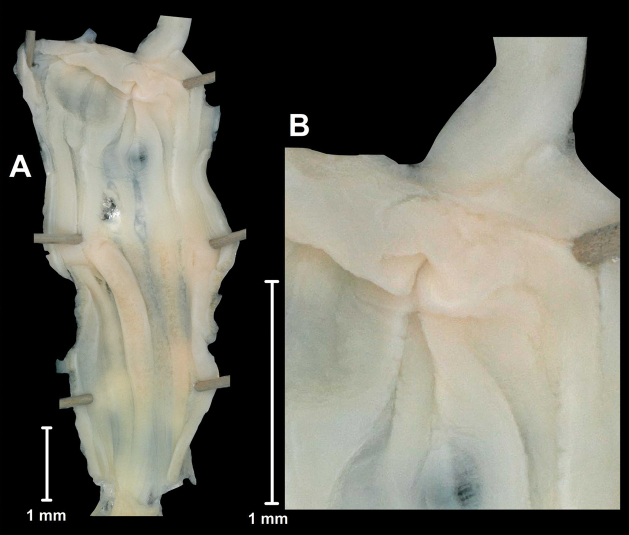
Inner wall of the penis of *Bouchetcamaenaplatytropis* Möllendorff, 1894 (ZMH 51934) **A** entire penis **B** penis-epiphallus transition.

**Figure 5.**
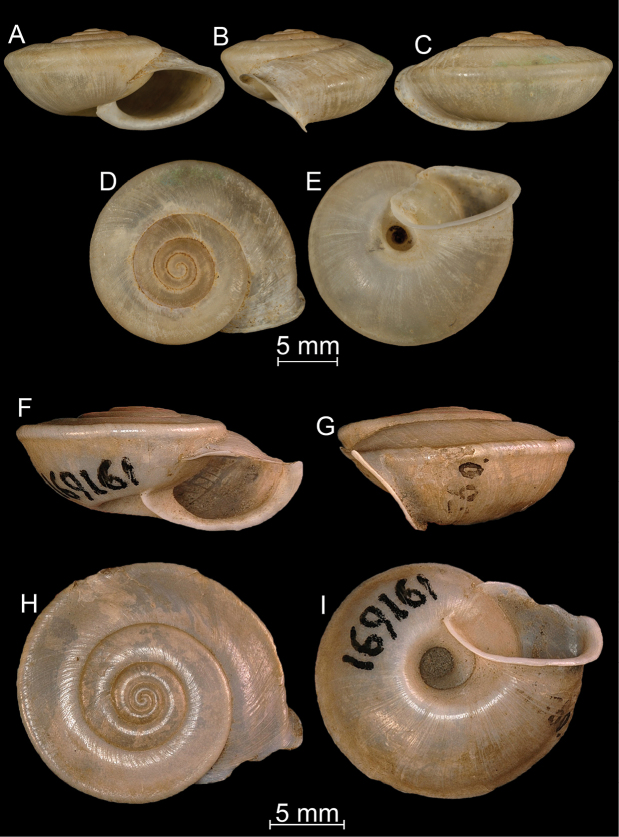
Shells of *Bouchetcamaena* species **A–E***Bouchetcamaenahuberi* Thach, 2018 (synonym of *B.fouresi* (Morlet, 1886), type species of *Bouchetcamaena*) **F–I***Bouchetcamaenagabata* (Gould, 1844) (MCZ Mala 169161). Photos: M. Caballer, MNHN (**A–E**) and downloaded from MCZ website (**F–I**).

We examined the reproductive anatomy of a specimen of *Chloritisplatytropis* Möllendorff, 1894 from Thailand (Figs 2A, 3, 4), which is conchologically similar to the type species of *Bouchetcamaena* Thach, 2018 (*Bouchetcamaenahuberi* Thach, 2018 [Fig. 5A]: synonym of *Helixfouresi* Morlet, 1886). Consequently, the anatomy of *Chloritisplatytropis* can be used to characterize *Bouchetcamaena*. *Chloritisplatytropis* differs from the *C.delibrata*-group only in the keeled shell, whereas their reproductive anatomy is similar. Moreover, *Chloritisgabata* (Gould, 1844) (Fig. 5B), which was described from Dawei, Myanmar (the type locality of Chloritisdelibratavar.procumbens), is similar to the type species of *Bouchetcamaena* in shell shape, size and sculpture (including the prominent keel), and differs from the *C.delibrata*-group only in the presence of a keel on the body whorl. We here move *Chloritisgabata* (Gould, 1844) and *Chloritisplatytropis* to the genus *Bouchetcamaena*, comb. nov.

Thus, *Bouchetcamaena* can host the entire Chloritisdelibrata -group with the exception of var. fasciata, which is transferred to *Burmochloritis* Godwin-Austen, 1920, comb. nov. due to the multiple narrow spiral bands on its shell. The anatomical characters are summarized in Table 1.

## ﻿Materials and methods

Determination of the number of shell whorls (precision to 0.25 whorl) follows Kerney and Cameron (1979: p. 13). In the case of close-up images, multilayer close-up photographs were taken of each shell.

**Figure 6.**
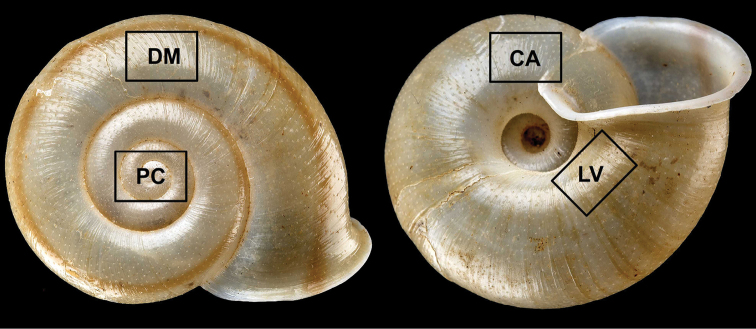
Positions of close-up images. Abbreviations: CA: callus; DM: middle of dorsal side; LV: last whorl; PC: protoconch. Not to scale.

Locality data presented with the specimen examined data are cited as verbatim from the specimen labels. For Indian and Burmese localities see Páll-Gergely et al. (2015a, 2015b). Measurements were taken on the visually selected largest and smallest specimens. Figure 6 presents the exact position of shell details that have to be checked when identifying species of *Bouchetcamaena*.

### ﻿Abbreviations

**BSNH**Boston Society of Natural History (Boston, USA)

**D** Shell diameter

**H** Shell height

**MCZ**Museum of Comparative Zoology (Cambridge, USA)

**NHM** The Natural History Museum (London, UK)

**NHMUK** when citing lots deposited in the NHM

**SMF**Senckenberg Forschungsinstitut und Naturmuseum (Frankfurt am Main, Germany)

**UMZC** University Museum of Zoology (Cambridge, UK)

**USNM**Smithsonian National Museum of Natural History (Washington, D.C., USA)

**ZSI**Zoological Survey of India (Kolkata, India)

## ﻿Taxonomy and systematics

### Camaenidae Pilsbry, 1895

#### ﻿Genus Bouchetcamaena

Taxon classificationAnimaliaStylommatophoraCamaenidae

Thach, 2018

F9CF6D4F-5CC3-51C8-B413-8AD40D994170

Bouchetcamaena Thach, 2018: 65.

##### Type species.

*Bouchetcamaenahuberi* Thach, 2018, by original designation (synonym of *Helixfouresi* Morlet, 1886 – see Páll-Gergely et al. 2020).

##### Diagnosis.

The shell characters are similar to those of most other *Chloritis*-like groups. Shell depressed to depressed globular (sometimes with a sunken apex), body whorl rounded, colour uniform with a single peripheral band, shell surface covered by hair scars (pits) of variable density (in some cases these are more or less absent on the last whorl) and deciduous periostracum of variable thickness, aperture rounded to oval/subrectangular, peristome expanded, parietal callus only indicated, umbilicus relatively narrow (narrower than one fourth of the shell’s width).

The genital organs [based on *B.procumbens* (Stoliczka 1871) and *B.platytropis* (this study)] are characterized by an elongated penis (spindle-shaped or with slightly swollen proximal end), the absence of a penial verge, a slender, cylindrical epiphallus longer than the penis, a retractor muscle inserted at the penis-epiphallus transition or on the distal end of the epiphallus, and a slender, pointed, elongated flagellum.

##### Remarks.

We only move the few species revised here to this genus. However, several other camaenid species from Southeast Asia may belong to *Bouchetcamaena*, which will be revealed by future studies.

#### ﻿ Bouchetcamaena delibrata

Taxon classificationAnimaliaStylommatophoraCamaenidae

(Benson, 1836) comb. nov.

04EE659C-5149-5852-AE06-B5D44CE11682

**Figure 7.**
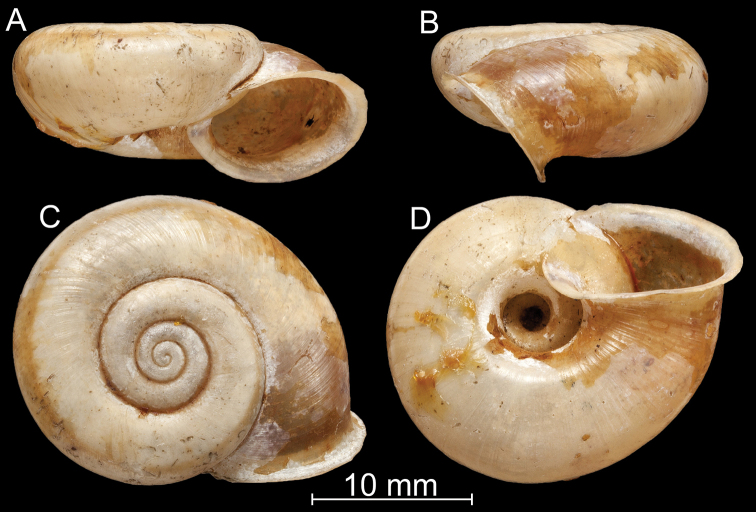
*Bouchetcamaenadelibrata* (Benson, 1836), comb. nov. UMZC 2387 (syntype).

**Figure 8.**
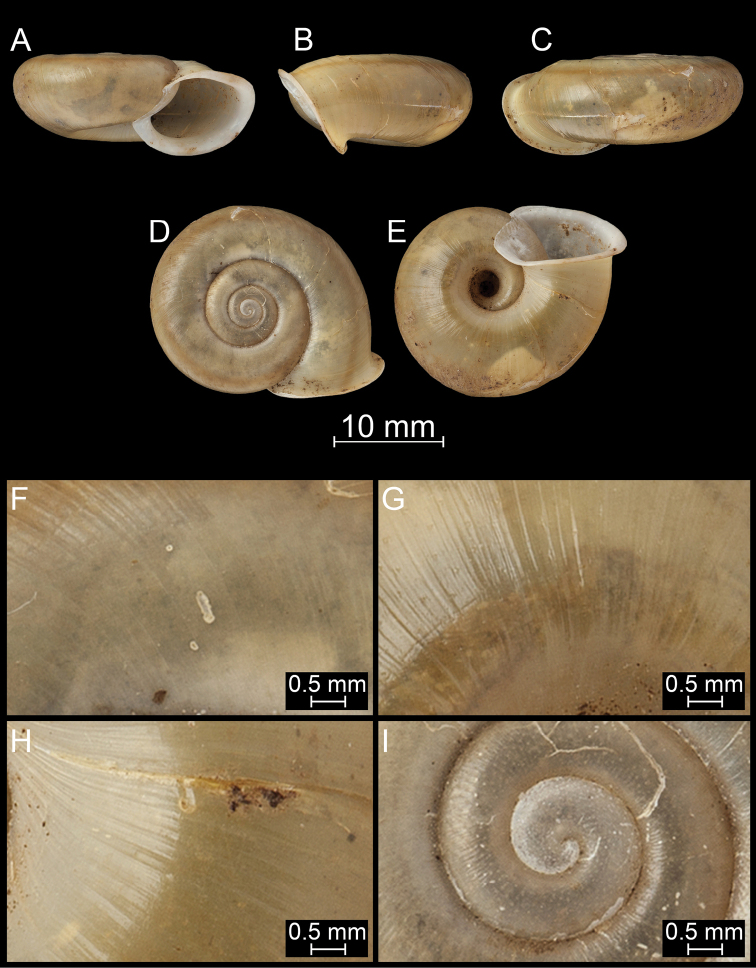
*Bouchetcamaenadelibrata* (Benson, 1836), comb. nov. NHMUK 1903.7.1.381. For positions of close-up images see Fig. 6 (F = DM, G = CA, H: LV, I: PC).

**Figure 9.**
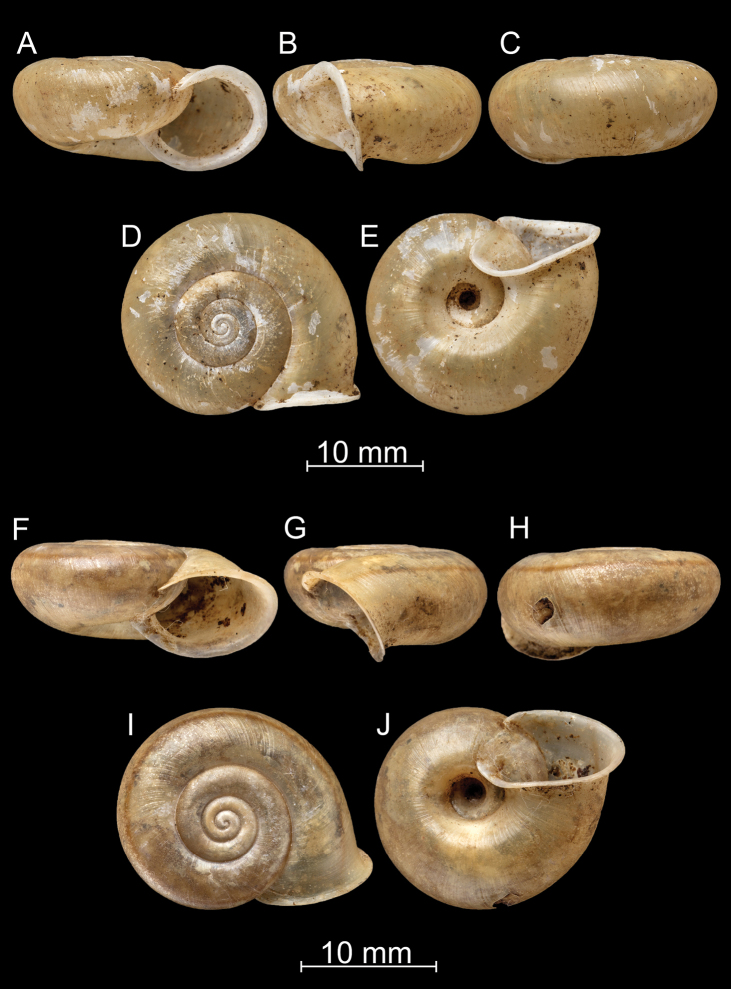
*Bouchetcamaenadelibrata* (Benson, 1836), comb. nov. **A–E**NHMUK 1903.7.1.61 **F–J** 1903.7.1.381.a.

**Figure 10.**
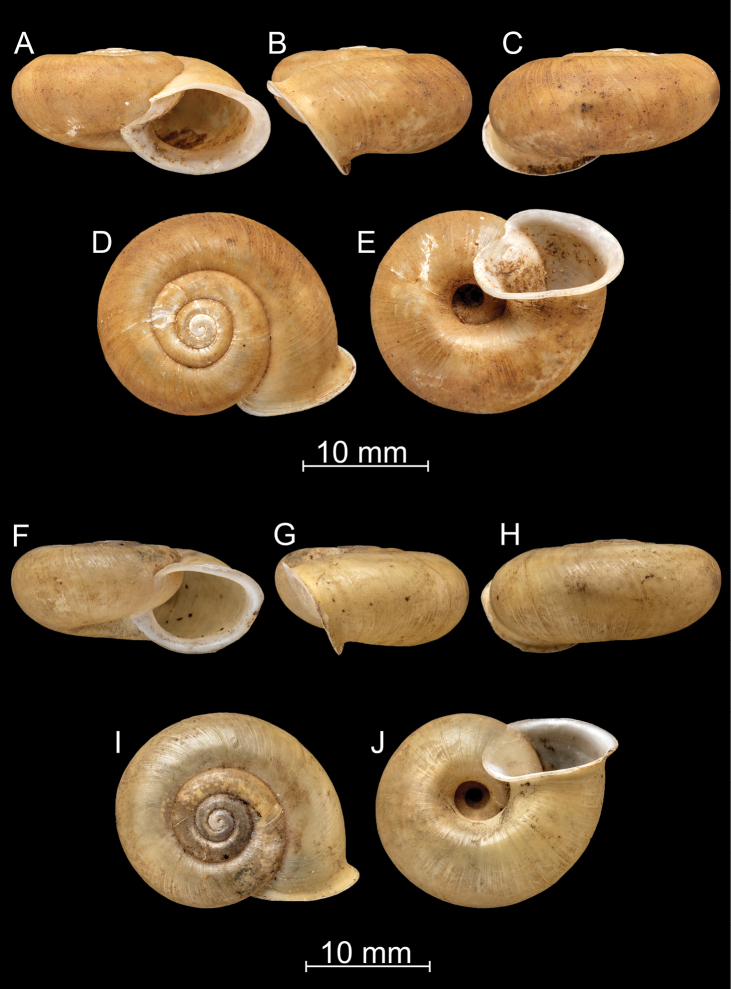
*Bouchetcamaenadelibrata* (Benson, 1836), comb. nov. **A–E**NHMUK 1920.1.28.12–13 **F–J**NHMUK 23-iii-15.

Figures 7 , 8 , 9 , 10

Helix delibratus Benson, 1836: 352.
Chloritis (Trichochloritis) delibrata Gude, 1914: 172.
Chloritis delibrata Richardson, 1985: 92.
Chloritis delibrata Ramakrishna et al., 2010: 326.

##### Type locality.

“North-East frontier of Bengal”.

##### Types examined.

UMZC 2387 (1 syntype).

##### Additional material examined.

Khasi Berge, coll. Möllendorff, SMF 27140/1; Hinterindien, coll. Möllendorff, SMF 27141 (2 shells, mixed sample with another species); India, NHMUK 1871.9.23.99/1 (1 shell, mixed lot with *B.foveata*: NHMUK 1871.9.23.99/2); Khasi Hills, blue label 7/12/06, NHMUK 20191138 (1 shell); Khasi Hills, coll. Godwin-Austen, NHMUK 1903.7.1.381/2 (1 shell, mixed lot with *B.fasciata*, NHMUK 1903.7.1.381/1, this is the syntype lot of *fasciata*); Khasi Hills, coll. Godwin-Austen, no. 183, NHMUK 1903.7.1.381a/1 (1 shell, mixed lot with *B.fusca*: NHMUK 1903.7.1.381a/2); Khasi Hills, NHMUK 1920.1.28.12-13/1 (1 shell, mixed lot with *B.foveata*: NHMUK 1920.1.28.12-13/2); Munipur, coll. Godwin-Austen, NHMUK 1903.7.1.391/1 (1 shell, mixed lot with *B.fusca*: NHMUK 1903.7.1.391/2); Sibsagar, Assam, coll. Godwin-Austen, NHMUK 1909.3.15.23 (2 shells); South Sylhet Hills, coll. W Chennell, NHMUK 1903.7.1.61/1 (1 shell, mixed lot with *B.subdelibrata*: NHMUK 1903.7.1.61/2).

##### Diagnosis.

Shell large, flat, olive green, glossy, last whorl practically without hair scars, or widely-spaced hair scars near the parietal callus, aperture oval.

##### Description.

Shell relatively large, rather thin-walled; depressed, dorsal side usually entirely flat, rarely very slightly elevated; colour greenish-olive to greenish-yellowish with an obscure, reddish band just above the blunt keel (rarely missing); protoconch consists of 1.75 whorls, with very fine radial ribs and regularly arranged hair scars (pits); entire shell with 4 whorls; separated by a moderately deep suture; teleoconch overall glossy, with irregular, fine growth lines, dorsal side of first 2.0–2.5 whorls covered by widely-spaced hair scars (= pits), and short hairs near suture; ventral side of body whorl without hair scars or widely spaced (in some specimens somewhat denser than in others) pits near the parietal callus only; aperture oval/subrectangular; peristome strongly expanded and slightly reflected in direction of umbilicus; palatal part with very thin, whitish, semi-transparent layer, showing hair scars on penultimate whorl; umbilicus open, relatively wide, funnel-shaped, peri-umbilical keel only very slightly indicated.

##### Measurements.

D = 21.4–24.4 mm, H = 9.2–10.3 mm (n = 4).

##### Differential diagnosis.

*Bouchetcamaenasubdelibrata* sp. nov. differs from *B.delibrata* mainly in the presence of hair scars on the entirety of the last whorl. For further differences, see under that species.

##### Distribution.

This species is apparently widespread in the southwestern Himalaya (Khasi Hills, Manipur, Silhet).

#### ﻿ Bouchetcamaena foveata

Taxon classificationAnimaliaStylommatophoraCamaenidae

Páll-Gergely sp. nov.

48269C97-A724-5E4B-B9D3-9E1093D8577E

http://zoobank.org/768A3926-C993-458D-8D27-CC787DCE6633

Figure 11

##### Type material.

***Holotype***: Khasia Hills [Meghalaya, India], 183, Assam, coll. Godwin-Austen, NHMUK 20191130/2 (D: 20.5 mm, 9.1 mm, mixed lot with *B.fasciatus*: NHMUK 20191130/1).

***Paratypes***: Assam, coll. C. Bosch ex coll. H. Rolle, SMF 297336 (2 paratypes, labelled as *delibrata*f.major); Assam: Chenapoongu, coll. Jetschin ex coll. Gude 1900, SMF 91157 (2 paratypes); Assam: Khasia Hills, coll. C. Bosch ex coll. H. Rolle ex coll. Schlüter, SMF 297335 (2 paratypes); (1) Khasi Hills, Assam, (2) Burma, A.S. Kennard coll., Acc. No. 1824, NHMUK 20191136/2 (2 paratypes, mixed lot with *B.procumbens*: NHMUK 20191136/1, the Khasi Hills probably refers to *foveata*, whereas Burma refers to *procumbens*); India, NHMUK 1871.9.23.99/2 (1 paratype, mixed lot with *B.delibrata*: NHMUK 1871.9.23.99/1); India, Laity (?) valley, H.F./W.T. Blanford coll., acc. 1944, NHMUK 20191135 (2 paratypes); Khasi Hills, blue label, 13/II/00, NHMUK 20191140 (2 paratypes, shells corroded by Byrne’s disease); Khasi Hills, NHMUK 1920.1.28.12-13/2 (1 paratype, mixed lot with *B.delibrata*: NHMUK 1920.1.28.12–13/1); Nemotha, blue label, 7/3/91, NHMUK 20191139 (2 paratypes).

##### Diagnosis.

Shell relatively large, fragile, thin-walled, dorsal side flat or even slightly sunken, colour light yellow to whitish, with a faint peripheral band; hair scars represented as elevated knobs (like strawberry seeds), or even hair scars represented as truncated hairs or short, slender, pointed hairs; aperture oval, umbilicus relatively narrow.

**Figure 11.**
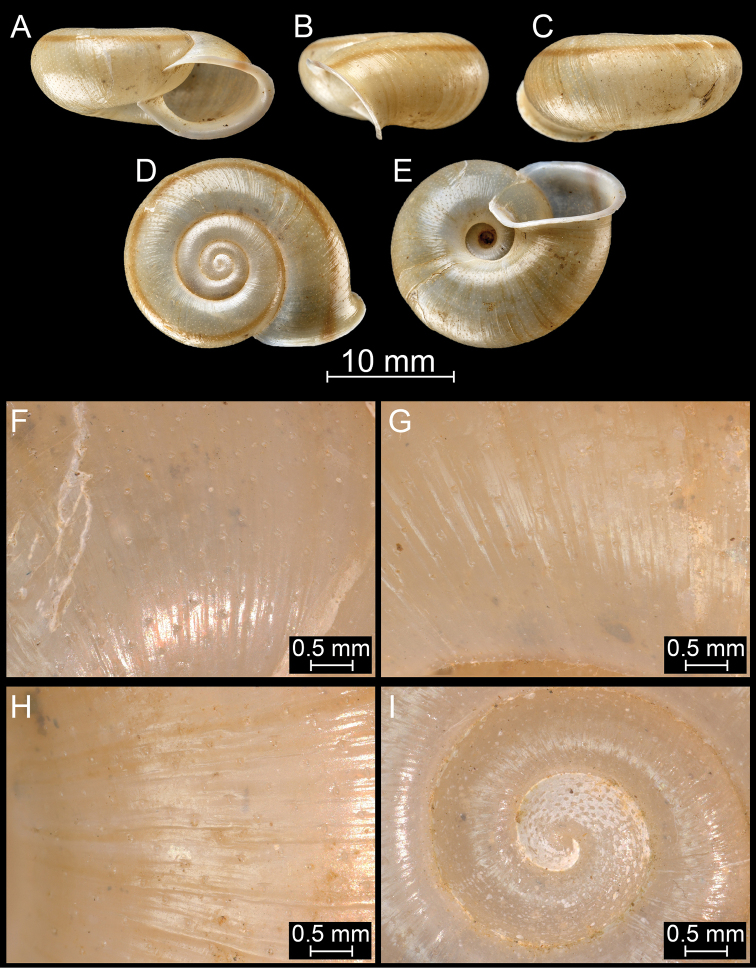
*Bouchetcamaenafoveata* Páll-Gergely sp. nov., holotype (NHMUK 20191130/2). For positions of close-up images see Fig. 6 (F = DM, G = CA, H: LV, I: PC).

##### Description.

Shell medium-sized to large, thin-walled; dorsal side flat or even sunken; basic colour light yellowish to whitish, a peripheral band of various thickness present in all specimens, running around the shoulder or the body whorl; protoconch consisting of 1.50–1.75 whorls, finely wrinkled and covered by widely-spaced hair scars reminiscent of strawberry seeds; entire shell consisting of slightly less or more than 3.75–4 whorls, separated by a relatively deep suture; teleoconch very finely and irregularly wrinkled; hair scars (reminiscent of strawberry seeds) widely-spaced, clearly visible on the entire teleoconch; occasionally (near suture, behind expanded peristome, inside umbilicus, etc.) short, slender, pointed hairs remaining; hairs inside umbilicus denser than elsewhere on the teleoconch; aperture oval/subrectangular; peristome strongly expanded and slightly reflected, especially in direction of umbilicus; palatal part with thin, whitish, semi-transparent layer, which allows hair scars of penultimate whorl to be seen; umbilicus open, normally wide, funnel-shaped, peri-umbilical keel blunt.

##### Measurements.

D = 20.3–20.5 mm, H = 9.1–10.5 mm (n = 3).

##### Differential diagnosis.

This new species differs from that which is the most similar, *B.delibrata*, in having a flatter dorsal side, glossier shell, and deep hair scars on the entire surface. The hair scars of *B.subdelibrata* sp. nov. are much finer and denser on the entire shell surface.

##### Etymology.

The new species is named after its conspicuously pitted (= *foveatus* in Latin) surface.

##### Distribution.

All samples with relatively precise localities were collected in the Khasi Hills.

#### ﻿ Bouchetcamaena fusca

Taxon classificationAnimaliaStylommatophoraCamaenidae

Páll-Gergely sp. nov.

0CE5D6A5-99C4-5004-ADB7-879A36D61408

http://zoobank.org/75255BD2-CBD1-4BA5-B0C0-82DD4E88F5CE

Figure 12

##### Type material.

***Holotype***: Munipur [India, Manipur], coll. Godwin-Austen, NHMUK 1903.7.1.391/2 (D: 16.8 mm, H: 8 mm, mixed lot with *B.delibrata*, NHMUK 1903.7.1.391/1).

***Paratypes***: same data as holotype, NHMUK 1903.7.1.391/3 (1 shell: paratype); Gaziphima, Naga Hills, Munipur frontier line, coll. Godwin-Austen, NHMUK 1903.7.1.385 (2 paratypes); Khasi Hills, coll. Godwin-Austen, no. 183, NHMUK 1903.7.1.381a/2 (1 shell, mixed lot with *B.delibrata*, NHMUK 1903.7.1.381a/1); Manipur, coll. Godwin-Austen, NHMUK 20191134 (1 shell).

##### Diagnosis.

Shell small to medium-sized, with flat dorsal side or very slightly elevated spire; thick, brown, matt periostracum makes hair scars practically invisible, aperture oval.

##### Description.

Shell small to medium-sized; depressed-globular, dorsal side flat or spire very slightly elevated; body whorl slightly or relatively strongly but bluntly shouldered; colour brownish due to thick, matt periostracum; protoconch consists of 1.5 whorls, finely wrinkled, with short hairs near suture; in some specimens wrinkles only visible in the middle of whorls, whereas in others hair scars (pits) are also discernible; entire shell consisting of 3.75–4 whorls, suture moderately deep; short hairs visible in the suture and inside umbilicus; hair scars practically invisible due to thick periostracum; in one paratype (NHMUK 1903.7.1.391) periostracum of lighter colour around each hair scar on the ventral side, making the density of scars visible; very few hair scars visible at the parietal callus, but to a much lesser degree than in other species; aperture oval/subrectangular; peristome expanded and slightly reflected in direction of umbilicus; palatal part with a very thin, whitish, semi-transparent layer; hair scars not visible beneath parietal callus; umbilicus open, relatively narrow, peri-umbilical keel only very slightly indicated.

**Figure 12.**
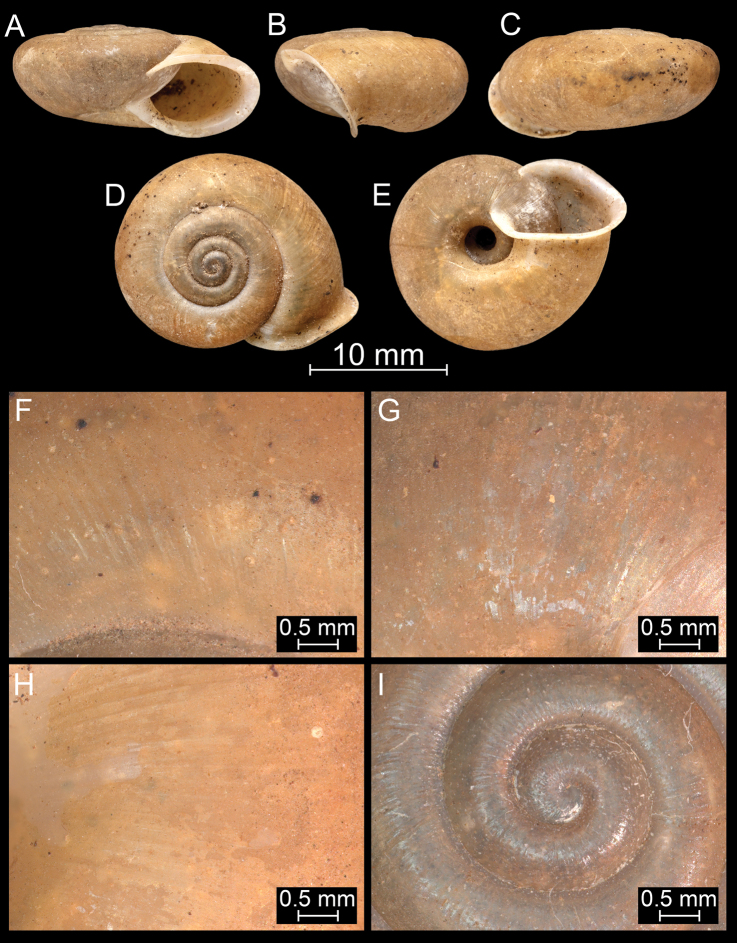
*Bouchetcamaenafusca* Páll-Gergely sp. nov., holotype (NHMUK 1903.7.1.391/2). For positions of close-up images see Fig. 6 (F = DM, G = CA, H: LV, I: PC).

##### Measurements.

D = 15.3–18.7 mm, H = 7.8–8.8 mm (n = 5).

##### Differential diagnosis.

*Bouchetcamaenararipila* sp. nov. is most similar to *B.fusca* sp. nov. in having a relatively small shell, narrow umbilicus and brown periostracum, but it differs in the strong, sparsely standing hair scars. All other *Bouchetcamaena* species have larger, lighter-coloured shells and a wider umbilicus.

##### Etymology.

The new species is named after its dark (*fuscus* in Latin) periostracum.

##### Distribution.

Seems to be restricted to Manipur, the Khasi and the Naga Hills (India).

#### ﻿ Helix (Trachia) delibratavar.khasiensis

Taxon classificationAnimaliaStylommatophoraCamaenidae

Nevill, 1877, taxon inquirendum

684CF16C-1B55-500C-AE16-A19ADD467913

Helix delibrata (?) var. Hanley & Theobald, 1870: pl. 14, fig. 9.
Helix (Trachia) delibrata Var. khasiensis Nevill, 1877: 21.
Chloritis (Trichochloritis) delibrata var. khasiensis Gude, 1914: 173.
Chloritis delibrata var. khasiensis Richardson, 1985: 93.
Chloritis delibrata khasiensis Ramakrishna et al., 2010: 326.

##### Type locality.

“Khasi Hills”.

##### Types.

Not found in the NHM or in the ZSI (Sheikh Sajan, pers. comm. April 2020).

##### Remarks.

Nevill (1877) named the form illustrated in Hanley and Theobald’s (1870) Conchologia Indica (pl. 14, fig. 9) as var. khasiensis, and mentioned that it has more raised and rounded whorls and a less open umbilicus than the typical *H.delibrata*. Nevill’s (1877) description was as follows: “The raised and rounded whorls, less open umbilicus, and contracted aperture will distinguish the form; it has sometimes a single brown band, but is often without it; it is tolerably abundant in the Naga and Khasi Hills. (...) Var. khasiensis, from Khasi Hills, axis 8.5, diam. 19.5 (apert. 9, diam 10.5) mm”.

*Bouchetcamaenafusca* sp. nov. agrees with some parts of Nevill’s description (more raised spire and narrower umbilicus than in other similar species), and the Naga and Khasi Hills also match, but the specimens we described above as *B.fusca* sp. nov. possessed no band and are all smaller than the size mentioned by Nevill. Furthermore, the shell illustrated by Hanley and Theobald (1870) possesses a subsutural furrow (a furrow running between the middle of the body whorl and the suture), which is absent in all *B.fusca* sp. nov. shells we examined. Thus, we decided to describe these specimens as a new species and consider the name Helix (Trachia) delibrata var. khasiensis as a taxon inquirendum.

#### ﻿ Bouchetcamaena platytropis

Taxon classificationAnimaliaStylommatophoraCamaenidae

(Möllendorff, 1894) comb. nov.

E31DF58F-032D-5606-BEE0-D71033F3C7BE

Figures 2 , 3 , 4

Chloritis platytropis Möllendorff, 1894: 150.
Chloritis (Trichochloritis) platytropis platytropis Zilch, 1966: 304, pl. 10, fig. 34.
Chloritis platytropis platytropis Maassen, 2001: 121.

##### Types examined.

Siam, Tschaya, coll. O. Möllendorff ex coll. Roebelen, SMF 8526/1 (lectotype of *Chloritisplatytropis*); same data, SMF 8527/1 (paralectotype of *Chloritisplatytropis*); Golf von Siam, Insel Samui, coll. O. Möllendorff ex coll. Roebelen, SMF 8524/1 (lectotype of Chloritisplatytropisvar.samuiana); same data, SMF 8525/1 (paralectotype of Chloritisplatytropisvar.samuiana).

##### Additional material examined.

Thailand, Phangnga: Thai Mueang: Ton Prai waterfall, 90 m, 08°26'11"N, 098°18'33"E, leg. Hausdorf, 02.08.2010, ZMH 51934 (1 dry shell + ethanol-preserved body).

##### Description of the genitalia.

Penis long, cylindrical, with swollen proximal part connected to adjacent epiphallic area by weak fibres; internally with ca. 6 wide, longitudinal, low folds; no penial verge present, although the folds form a circular ring with slightly elongated margin; epiphallus slightly longer than penis; retractor muscle slender, inserting on the distal end of epiphallus near its joint with penis; flagellum long, slender, pointed; no penial caecum present; vagina shorter and thicker than penis; spermoviduct elongated, stalk of bursa copulatrix very long, with some central swelling, bursa small, rounded; albumen gland long, banana-shaped, talon small.

#### ﻿ Bouchetcamaena procumbens

Taxon classificationAnimaliaStylommatophoraCamaenidae

(Gould, 1844) comb. nov.

89FE017E-D906-5EE8-81AE-B9E99EDF3FDD

**Figure 13.**
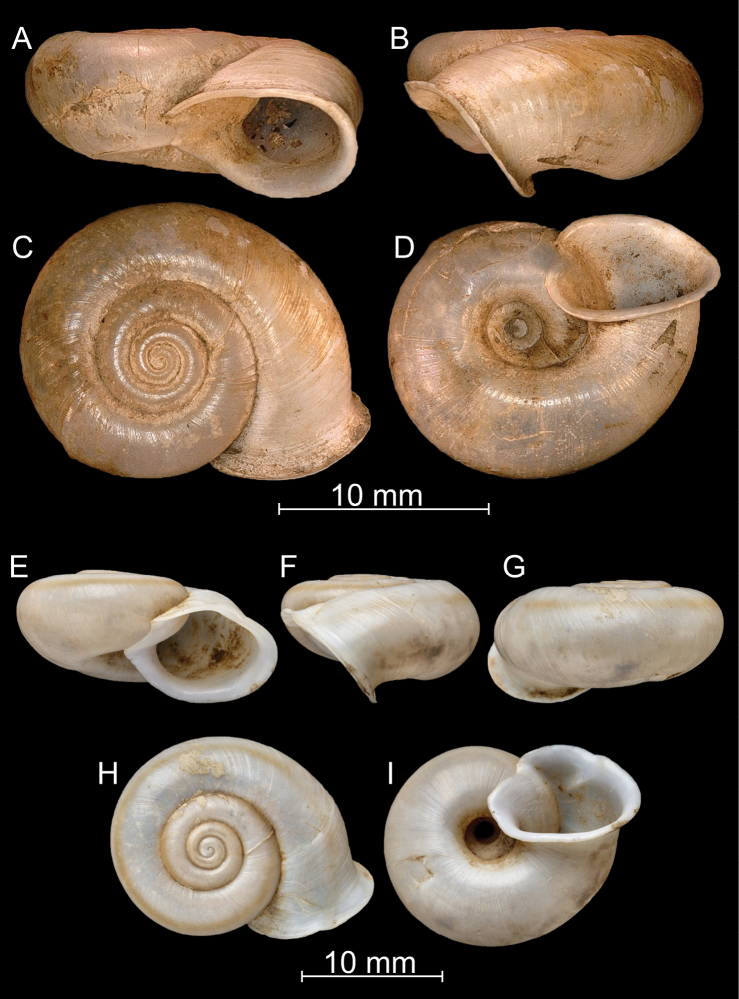
*Bouchetcamaenaprocumbens* (Gould, 1844), comb. nov. **A–D** lectotype (MCZ 169311) **E–I**NHMUK 1862.11.19.12.

**Figure 14.**
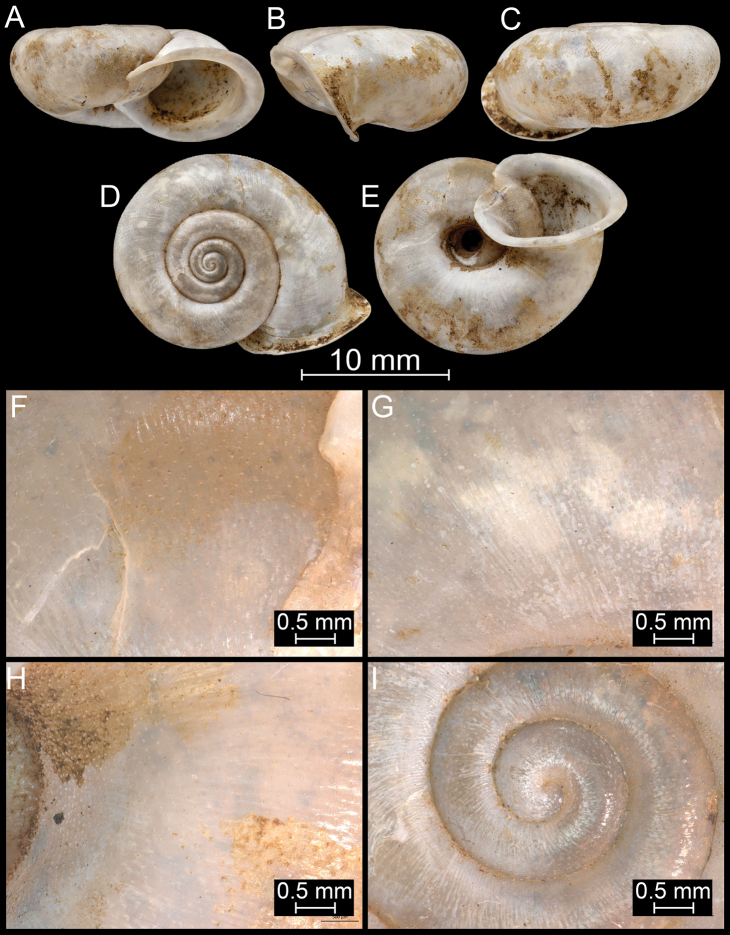
*Bouchetcamaenaprocumbens* (Gould, 1844), comb. nov. NHMUK 1903.7.1.386. For positions of close-up images see Fig. 6 (F = DM, G = CA, H: LV, I: PC).

**Figure 15.**
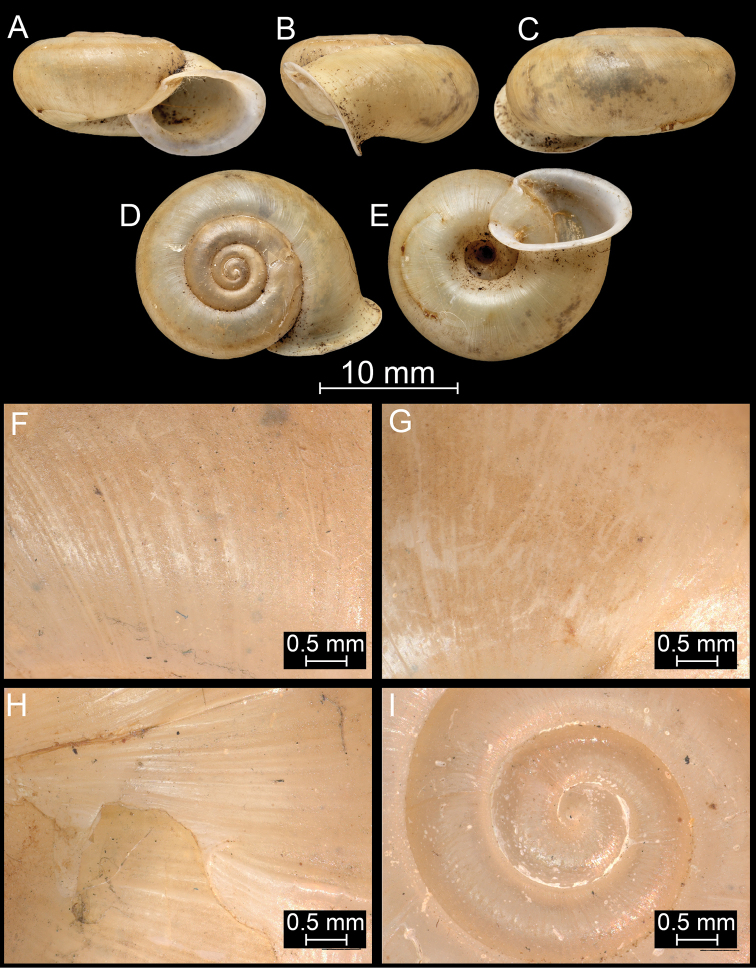
*Bouchetcamaenaprocumbens* (Gould, 1844), comb. nov. NHMUK 1906.2.2.121. For positions of close-up images see Fig. 6 (F = DM, G = CA, H: LV, I: PC).

Figures 13 , 14 , 15

Helix procumbens Gould, 1844: 453, pl. 24, fig. 1.
Helix delibrata Hanley & Theobald, 1870: pl. 14, fig. 10.
Chloritis (Trichochloritis) delibrata var. procumbens Gude, 1914: 172–173.
Trachia delibrata f. procumbens Stoliczka, 1871: 225, pl. 16, figs 1–3 (reproductive anatomy, jaw, radula).
Chloritis delibrata var. procumbens Richardson, 1985: 93.

##### Type locality.

“Province of Tavoy in British Burmah” (from the title).

##### Types examined.

Tavoy, British Burmah, leg. F. Mason, MCZ 169311 (lectotype: labelled as holotype, see remarks).

##### Additional material examined.

**white type**: (1) Khasi Hills, Assam, (2) Burma, A.S. Kennard coll., Acc. No. 1824, NHMUK 20191136/1 (1 shell, mixed lot with *B.foveata*: NHMUK 20191136/2; the Khasi Hills material probably refers to *foveata*, whereas the Burma material refers to *procumbens*); India, “Khasi Hills”, NHMUK 1862.11.19.12 (2 shells); Moulmain, Tenasserim, coll. Stoliczka, NHMUK 1903.7.1.387 (1 shell); Pegu, ex coll. Godwin-Austen, NHMUK 1903.7.1.386 (1 shell); Pegu, NHMUK 1888.12.4.1114–1115 (2 shells); Tavoy, Burmah, Museum Cuming, NHMUK 20191137 (1 shell). **darker type**: Arakan, coll. H.F. Blanford, NHMUK 1909.3.15.25 (1 shell); Mutan, Tenasserim, coll. Godwin-Austen, NHMUK 20191133 (1 shell); Pegu, Arakan Hills, NHMUK 1906.2.2.121 (4 shells, one of them with widely-spaced pits, others with hardly visible scars, see remarks).

##### Diagnosis.

Shell small to medium-sized, with flat dorsal side or slightly sunken or very slightly elevated spire; aperture oval, peristome strongly expanded, umbilicus narrow and deep; sculpture variable: in some shells the thick, brown, matt periostracum makes hair scars practically invisible, in other shells the hair scars (pits) are usually densely arranged on the entire shell.

##### Description.

Shell medium-sized, rather thin-walled; dorsal side flat, very rarely (NHMUK 1906.2.2.121) slightly domed; shell shape “nautiliform” (i.e., initial whorls closely coiled, body whorl conspicuously expanded); colour whitish to light yellowish with somewhat darker yellowish, matt (dull) periostracum in some places, or, as in one sample (Mutan, Tenasserim), on the entire shell; a normally wide, faint reddish-brown belt running around the shoulder of the body whorl (can be entirely absent); protoconch consisting of 1–1.5 whorls, finely wrinkled and covered by hair scars (pits) or small, mamilla-like hairs; entire shell consisting of 3.75–4.75 whorls, separated by a deep suture; teleoconch finely and irregularly wrinkled; sculpture variable: in typical shells some darker yellowish-light brownish periostracum covers parts of the teleoconch, and the densely arranged hair scars are only visible in the suture on the dorsal side and near the parietal callus on the ventral side; in the “pitted form”, hair scars densely arranged on entire shell and clearly visible on both the dorsal and ventral sides; aperture oval/subrectangular; peristome strongly expanded and slightly reflected in direction of umbilicus; palatal part with a thin, whitish, semi-transparent layer, with hair scars visible on penultimate whorl; umbilicus open, narrow, funnel-shaped, peri-umbilical keel blunt.

##### Measurements.

D = 15.8–19.5 mm, H = 7.8–9.4 mm (n = 7).

##### Differential diagnosis.

The smaller shell size and the more reflected peristome distinguish this species from the most similar species, *B.delibrata*. Furthermore, the “white type” *B.procumbens* differs from *B.delibrata* in the presence of dense and prominent hair scars.

##### Distribution.

Seems to be restricted to southern Myanmar.

##### Remarks.

Johnson (1964) mentioned that the figured “holotype” (MCZ 169311) is from the NYSM 232 (original no. A 567), and that there is a “paratype” (USNM 611226) from the same NYSM lot. Johnson’s listing of the specimen as a “holotype” has to be accepted as an indirect lectotype designation by inference of holotype (see ICZN Art. 74.6). Furthermore, two additional “paratypes” (= paralectotypes) are found in the MCZ (reg. no.: 87935, ex BSNH).

There are two forms of this species. One is lighter in colour, some of the specimens have a slightly sunken spire and possess uniformly arranged, strong hair scars on the entire surface of the shell (“white type”), whereas the other type has a darker shell (yellowish to light brownish) with hardly visible hair scars (“darker type”). The sample NHMUK 1906.2.2.121 contains four shells of identical appearance; however, one of them has widely-spaced pits, whereas the other three have only some pits on the callus area but otherwise no hair scars. Thus, it seems that the strength of the hair scars is variable within this species, unlike in all other species of this group. Since the shell and aperture shape are, with the exception of spire height, practically identical, and there are transitional forms between the two types in terms of shell sculpture, we provisionally treat them as one species. There are no clearly visible hair scars on the lectotype. Thus, the form without prominent hair scars is considered typical *procumbens*, and the “pitted form” is considered atypical.

#### ﻿ Bouchetcamaena raripila

Taxon classificationAnimaliaStylommatophoraCamaenidae

Páll-Gergely sp. nov.

3A0F1625-EDF5-55D8-A84B-807BDA15FD13

http://zoobank.org/CE6219D5-7470-43D8-BDC2-D07137D2ED37

Figure 16

##### Type material.

***Holotype***: Kopanedza, coll. Godwin-Austen, NHMUK 20191131 (D: 15.4 mm, H: 8.1 mm).

##### Diagnosis.

Shell small, with slightly elevated spire; periostracum thick, brown, hair scars (truncated hairs or strawberry seed-like scars) extremely sparsely arranged on the body whorl; aperture oval, almost rounded.

##### Description.

Shell small; depressed-globular, with very slightly elevated spire (low domed dorsal side); body whorl rounded; colour brown due to thick, matt (dull) periostracum, locally worn locally making the nude whitish shell surface visible; protoconch consists of 1.5 whorls, finely wrinkled, with hair scars reminiscent of strawberry seeds; entire shell consisting of 4.25 whorls, suture moderately deep; inside of umbilicus and suture with mamilla-like, relatively densely arranged hair scars; other parts of teleoconch with extremely widely-spaced hairs (truncated, reddish-brown hairs or strawberry seed-like scars, and very few, relatively long, conical hairs); aperture oval, almost rounded; peristome strongly expanded and slightly reflected in direction of umbilicus; palatal part with a very thin, whitish, semi-transparent layer, which allows hair scars of the penultimate whorl to be seen; umbilicus open, narrow, funnel-shaped, peri-umbilical keel blunt.

**Figure 16.**
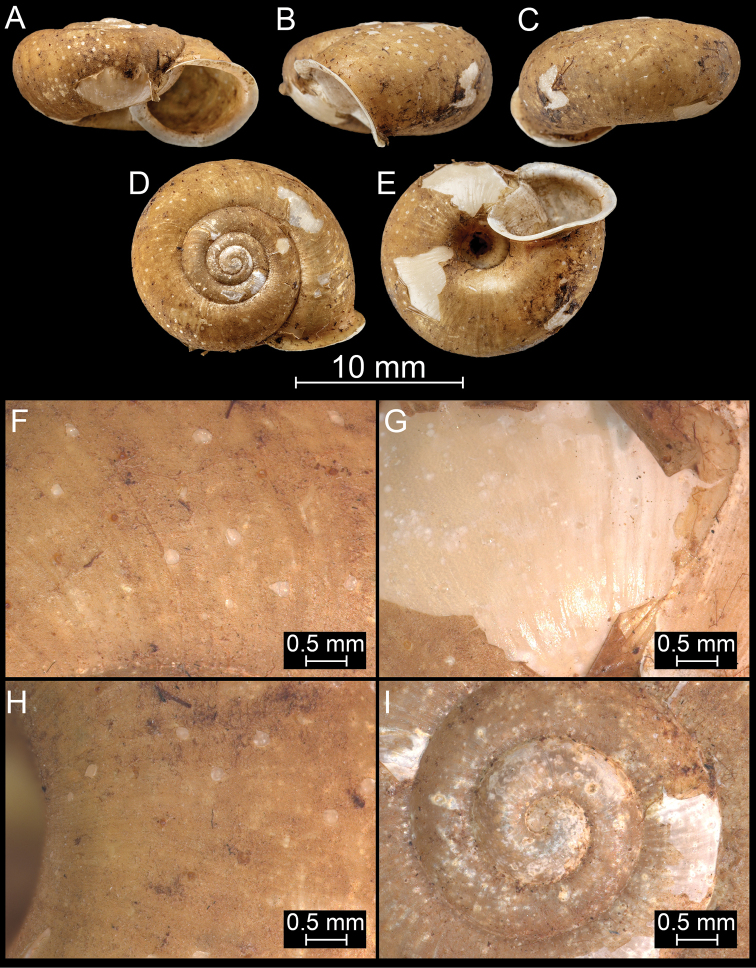
*Bouchetcamaenararipila* Páll-Gergely sp. nov., holotype (NHMUK 20191131). For positions of close-up images see Fig. 6 (F = DM, G = CA, H: LV, I: PC).

##### Measurements.

D = 15.4 mm, H = 8.1 mm (n = 1).

##### Differential diagnosis.

Differs from *B.procumbens* by having a more rounded shell shape and the last whorl is also more rounded. Most important are the extremely widely-spaced and prominent hair scars. This latter trait distinguishes *B.raripila* sp. nov. from all other similar species.

##### Etymology.

The name *raripila* refers to the few hairs/hair scars on the shell surface (*rarus*: few, *pilus*: hair in Latin).

##### Distribution.

The new species is known from the type locality only. Kopanedza (also spelled Kopamedza) is situated in the Barail Range, Dafla Hills (India), although its exact locality is unknown (Páll-Gergely et al. 2015b).

#### ﻿ Bouchetcamaena subdelibrata

Taxon classificationAnimaliaStylommatophoraCamaenidae

Páll-Gergely sp. nov.

F8A5BDC8-43DB-5EAE-946B-06B1E73BE401

http://zoobank.org/BADF67BD-2D56-40D2-8378-823B52F21352

Figure 17

##### Type material.

***Holotype***: S. Silhet, leg. Chennell, coll. Godwin-Austen, NHMUK 20191132/1 (D: 17.7 mm, 8.6 mm).

***Paratypes***: Same data as holotype, NHMUK 20191132/2 (1 paratype); Habiang, Garo Hills, coll. Godwin-Austen 183, ex coll. W. Blanford, NHMUK 1906.1.1.714 (3 paratypes); South Sylhet Hills, coll. W Chennell, NHMUK 1903.7.1.61/2 (1 paratype, mixed lot with *B.delibrata*: NHMUK 1903.7.1.61/1).

##### Additional material examined.

Same data as holotype, NHMUK 20191132/3 (7 juvenile shells).

##### Diagnosis.

Shell medium-sized, nearly flat, greenish, glossy, entire shell with densely arranged hair scars (mostly hardly visible), aperture oval, peristome not particularly expanded.

##### Description.

Shell medium-sized, rather thin walled; depressed, dorsal side entirely flat (type series), or slightly elevated (Habiang); colour greenish to dark yellowish with an obscure, reddish band just above the blunt keel; protoconch consisting of 1.5 whorls, with densely arranged, clearly visible, knob-like hair scars; entire shell with 3.50–3.75 whorls; separated by a rather deep suture; teleoconch overall glossy, with irregular, fine growth lines, ventral side and edge of body whorl (except for last quarter whorl) covered with densely-arranged hair scars, some hair scars also recognizable on the last quarter whorl, but not regular as on the preceding areas; last whorl of dorsal side dominated by wrinkles, and regular hair scars only visible in areas before the last half whorl; aperture almost rounded, slightly oval; peristome strongly expanded and slightly reflected in direction of umbilicus; palatal part with a very thin, whitish, semi-transparent layer, which allows hair scars on penultimate whorl to be seen; umbilicus open, normally wide, funnel-shaped, peri-umbilical keel blunt, only very slightly indicated.

**Figure 17.**
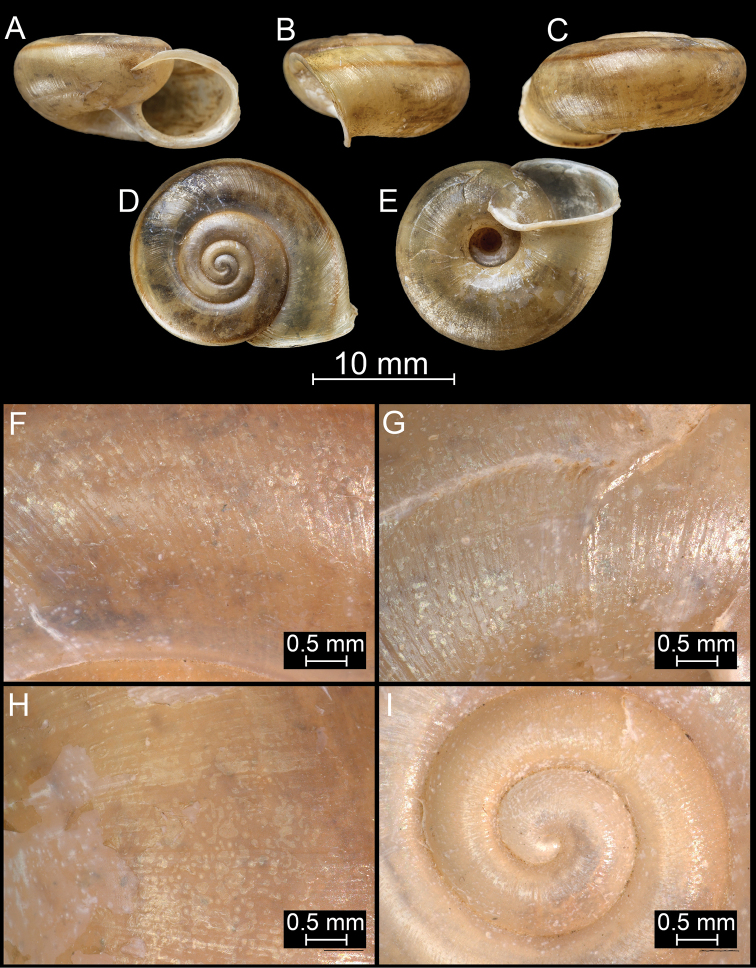
*Bouchetcamaenasubdelibrata* Páll-Gergely sp. nov., holotype (NHMUK 20191132). For positions of close-up images see Fig. 6 (F = DM, G = CA, H: LV, I: PC).

##### Measurements.

D = 17.7–19.3 mm, H = 8.6–9.9 mm (n = 4).

##### Differential diagnosis.

*Bouchetcamaenadelibrata* is most similar to the new species, but it is larger, has a more strongly depressed shell, elongated aperture and expanded peristome, and lacks hair scars on the last half whorl. The hair scars of *B.delibrata* are more widely-spaced than those of *B.subdelibrata* sp. nov.

##### Etymology.

The specific epithet refers to the most similar species.

##### Distribution.

The new species is known from the Garo Hills (Meghalaya, India), and the neighbouring Silhet Hills to the south.

#### ﻿Genus Burmochloritis

Taxon classificationAnimaliaStylommatophoraCamaenidae

Godwin-Austen, 1920

4BE4E1BB-E5D9-5815-98B3-1CB59FDA9711

Burmochloritis Godwin-Austen, 1920: 9.
Burmochloritis Schileyko, 2003: 1518.

##### Type species.

*Burmochloritiskengtungensis* Godwin-Austen, 1920, OD.

##### Remarks.

*Burmochloritis* Godwin-Austen, 1920 possesses a long flagellum, has penial caecum well-developed, and a large, additional organ originating from the wall of the vagina (Godwin-Austen 1920; and unpublished information). See also Table 1.

#### ﻿ Burmochloritis fasciata

Taxon classificationAnimaliaStylommatophoraCamaenidae

(Godwin-Austen, 1875) comb. nov.

6435E526-931B-5AAA-8E64-0DD74342AA10

Figure 18

Helix delibrata var. fasciata Godwin-Austen, 1975: 1, pl. 1, fig. 1.
Chloritis (Trichochloritis) delibrata var. fasciata Gude, 1914: 173.
Chloritis delibrata var. fasciata Richardson, 1985: 93.
Chloritis delibrata fasciata Ramakrishna et al., 2010: 326.

##### Type locality.

“On the high open grassy country of the West Khasi Hills”.

##### Types examined.

Khasi Hills, coll. Godwin-Austen, NHMUK 1903.7.1.381/1 (syntype, mixed lot with *B.delibrata*: NHMUK 1903.7.1.381/2).

##### Additional material examined.

Khasia Hills, 183, Assam, coll. Godwin-Austen, NHMUK 20191130/1 (1 shell, mixed lot with *B.foveata*: NHMUK 20191130/2).

##### Diagnosis.

Shell medium-sized, dorsal side domed, greenish-yellowish with a slender main spiral band and several thinner ones; last whorl with dense hair scars (pits) only near the parietal calls, aperture almost rounded.

##### Description.

Shell medium-sized, rather thick-walled; depressed globular, with slightly domed dorsal side; colour greenish-yellowish, with a main but still slender reddish band just above the blunt keel and several even thinner belts on both the dorsal and ventral surfaces; protoconch consists of 1.25 whorls, with very fine radial ribs and regularly arranged hair scars; entire shell consists of slightly more than 4 whorls, separated by a moderately deep suture; teleoconch covered with a matt periostracum, dense hair scars (and short hairs near suture) visible only near parietal callus; aperture rounded/subrectangular; peristome strongly expanded and slightly reflected in direction of umbilicus; parietal part with thin, whitish, semi-transparent layer, which allows hair scars of penultimate whorl to be seen; umbilicus open, relatively narrow, funnel-shaped, with blunt peri-umbilical keel.

**Figure 18.**
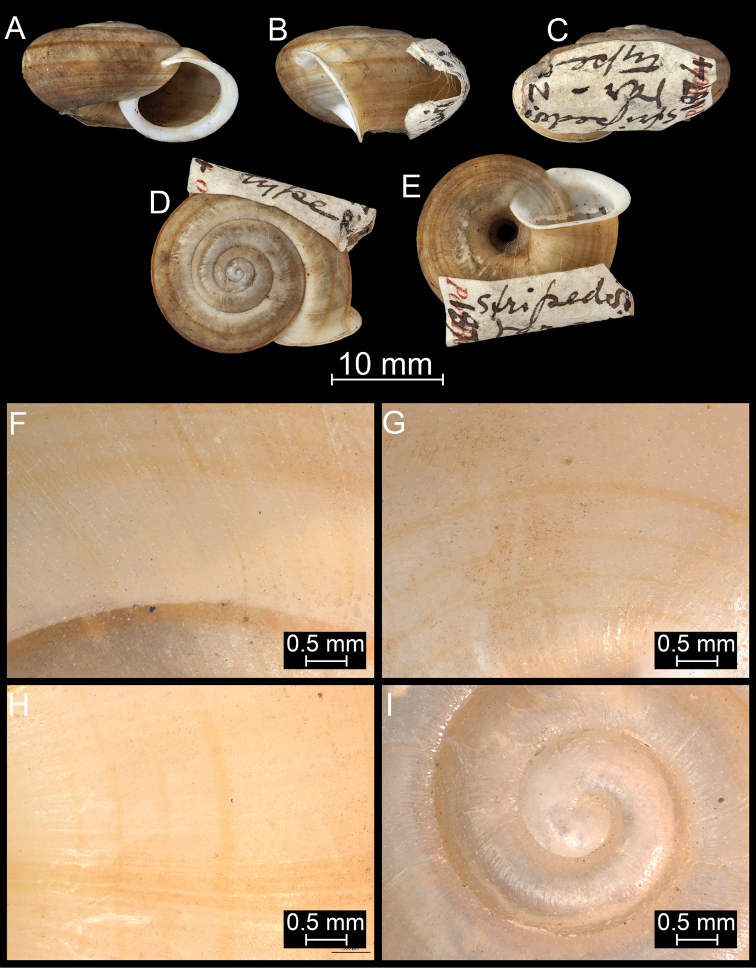
*Burmochloritisfasciata* (Godwin-Austen, 1875), comb. nov., syntype (NHMUK 1903.7.1.381/1). For positions of close-up images see Fig. 6 (F = DM, G = CA, H: LV, I: PC).

##### Remarks.

This species was described as a variety of *B.delibrata*, but differs in numerous shell characters (smaller, more globular shell, smaller protoconch, domed dorsal side, comparatively smaller, more rounded aperture, narrower umbilicus, denser hair scars). Thus, it should be handled as a species in its own right. Moreover, based on the multiple spiral bands (*Bouchetcamaena* species have no band or a single band), this species is transferred to the genus *Burmochloritis*, although this placement requires confirmation through anatomical examination.

## Supplementary Material

XML Treatment for Bouchetcamaena

XML Treatment for Bouchetcamaena delibrata

XML Treatment for Bouchetcamaena foveata

XML Treatment for Bouchetcamaena fusca

XML Treatment for Helix (Trachia) delibratavar.khasiensis

XML Treatment for Bouchetcamaena platytropis

XML Treatment for Bouchetcamaena procumbens

XML Treatment for Bouchetcamaena raripila

XML Treatment for Bouchetcamaena subdelibrata

XML Treatment for Burmochloritis

XML Treatment for Burmochloritis fasciata
